# Gut microbiome determines therapeutic effects of OCA on NAFLD by modulating bile acid metabolism

**DOI:** 10.1038/s41522-023-00399-z

**Published:** 2023-05-31

**Authors:** Jianjun Liu, Jiayi Sun, Jiangkun Yu, Hang Chen, Dan Zhang, Tao Zhang, Yicheng Ma, Chenggang Zou, Zhigang Zhang, Lanqing Ma, Xue Yu

**Affiliations:** 1 State Key Laboratory for Conservation and Utilization of Bio-Resources in Yunnan, School of Life Sciences, Yunnan University, Kunming, Yunnan 650091 China; 2 Yunnan Key Laboratory of Stem Cell and Regenerative Medicine, Institute of Biomedical Engineering, Kunming Medical University, Kunming, Yunnan 650500 China; 3 Department of Cardiology, Beijing Hospital, National Center of Gerontology, Institute of Geriatric Medicine, Chinese Academy of Medical Sciences & Peking Union Medical College, Beijing, 100730 China; 4 The First Affiliated Hospital, Yunnan Institute of Digestive Disease, Yunnan Clinical Research Center for Digestive Diseases, Kunming Medical University, Kunming, Yunnan 650032 China

**Keywords:** Metagenomics, Metagenomics

## Abstract

Non-alcoholic fatty liver disease (NAFLD), the most common chronic liver disease, had no approved pharmacological agents yet. Obeticholic acid (OCA), a novel bile acid derivative, was demonstrated to ameliorate NAFLD-related manifestations. Regarding the role of gut-liver axis in liver disease development, this study aimed to explore the potential role of gut microbiota in the treatment of OCA in NAFLD mice induced by the high-fat diet (HFD). Antibiotic-induced microbiome depletion (AIMD) and fecal microbiota transplantation (FMT) confirmed the critical role of gut microbiota in OCA treatment for NAFLD by effectively alleviating histopathological lesions and restoring liver function impaired by HFD. Metagenomic analysis indicated that OCA intervention in HFD mice remarkably increased the abundance of *Akkermansia muciniphila*, *Bifidobacterium* spp., *Bacteroides* spp., *Alistipes* spp., *Lactobacillus* spp., *Streptococcus thermophilus*, and *Parasutterella excrementihominis*. Targeted metabolomics analysis indicated that OCA could modulate host bile acids pool by reducing levels of serum hydrophobic cholic acid (CA) and chenodeoxycholic acid (CDCA), and increasing levels of serum-conjugated bile acids, such as taurodeoxycholic acid (TDCA) and tauroursodesoxycholic acid (TUDCA) in the HFD-fed mice. Strong correlations were observed between differentially abundant microbes and the shifted bile acids. Furthermore, bacteria enriched by OCA intervention exhibited much greater potential in encoding 7alpha-hydroxysteroid dehydrogenase (7α-HSDs) producing secondary bile acids rather than bile salt hydrolases (BSHs) mainly responsible for primary bile acid deconjugation. In conclusion, this study demonstrated that OCA intervention altered gut microbiota composition with specially enriched gut microbes modulating host bile acids, thus effectively alleviating NAFLD in the mice.

## Introduction

Non-alcoholic fatty liver disease (NAFLD) is a comprehensive clinical disease characterized by lipid accumulation in hepatocytes that encompasses simple steatosis, non-alcoholic steatohepatitis (NASH), and hepatic fibrosis and cirrhosis^1^. NAFLD is one of the most prevalent chronic liver disorders worldwide^2^, found in 10–40% of adults. In China, NAFLD is surrogating hepatitis B to become the predominant cause of chronic liver disorders^3^. NAFLD not only has a close relationship with insulin resistance but also potentially results from metabolic syndromes as well as obesity^4^. It is well-known that the gut-liver axis plays an important role in the pathogenesis of hepatic injuries like alcoholic liver disease, NAFLD, and hepatocellular carcinoma^5,6^. Despite the caloric restriction, body weight reduction, and the medicine for mitigating insulin resistance and hyperlipemia being considered effective strategies for NAFLD^7^, there are no accepted FDA-approved pharmacotherapies for treating or preventing NAFLD.

Gut microbiota had been discovered essential to human health^8^, and its crucial functions were confirmed by pre-clinical NAFLD/NASH models and NASH patients^9,10^. Usually, gut microbiota and their metabolites can induce fat accumulation and inflammation in the liver via the gut-liver axis^11^. However, the underlying pathogenesis of steatohepatitis mediated by gut microbiome is poorly understood. Recently, gut microbial signatures were implicated in the progression of human NAFLD, especially enriched bacteria of *Proteobacteria*, and the decreased abundance of *Rikenellaceae* and *Ruminococcaceae* in the gut of NAFLD patients^12^. Furthermore, gut commensal bacterial metabolites such as short-chain fatty acids, amino acids, and ethanol were involved in the pathogenesis of NAFLD^13,14^. So far, it was only proven to be effective in the clinical treatment of recurrent *C. diff* infection by fecal microbiota transplantation^15^. However, additional accumulating studies indicated the promising application of gut microbiota modulation for the treatment of other diseases, such as Autism spectrum disorders^16^, Ulcerative Colitis^17^, and immunotherapy-refractory melanoma^18^. Therefore, manipulating gut microbiota composition and replenishing commensal bacterial metabolites could be promising therapeutic approaches to NAFLD.

The chemical diversification of bile acids is the combined effort of both host and intestinal microbiota. Primary bile acids, such as CA and CDCA, are synthesized in hepatocytes via cytochrome P450-mediated oxidation of cholesterol in the liver^19^, and can be conjugated to either taurine (predominantly in mice) or glycine (mainly in humans) by bile acyl-CoA synthetase and bile acid-CoA:amino acid *N*‑acyltransferase to form taurocholic acid (TCA), taurochenodeoxycholic acid (TCDCA), glycocholic acid (GCA) and glycochenodeoxycholic acid (GCDCA)^20^. Conjugated primary bile acids are secreted from the liver into the bile canaliculus and gallbladder, and then released into the intestinal lumen by the gallbladder contracts in the postprandial state^21^, thus facilitating the emulsification and absorption of lipids in the small intestine^22^. Gut microbiota can modulate bile acid metabolism by encoding enzymes, such as BSHs, and hydroxysteroid dehydrogenases (HSDs). BSHs (classified as EC 3.5.1.24) are able to deconjugate both glycine- and taurine-bound primary bile acids, while HSDs are responsible for the biotransformation of primary bile acids into the secondary bile acids, especially 7α-HSDs (classified as EC 1.1.1.159) initiating the first step of oxidation of the hydroxyl groups in primary bile acids^23^. The farnesoid X receptor (FXR), one of the nuclear hormone receptor families, mainly expressed in the liver and intestine, can be activated by unconjugated bile acids (such as CDCA, Deoxycholic acid (DCA), and CA) which act as high-affinity ligand agonists^24^. FXR not only regulates bile acid synthesis and transport but also induces protective cellular responses in hepatic and gastrointestinal tissues and thereby regulates inflammation, immune responses, and liver regeneration^25,26^. Hence, gut microbiota is likely to regulate bile acid metabolism and further affect the host’s health status.

Obeticholic acid (OCA), the 6α-ethyl derivative of bile acids and potent activator of the FXR, was first approved as the therapeutic trial of primary biliary cholangitis^27^, also considered to treat NAFLD/NASH^28^. OCA could effectively alleviate fat accumulation in liver, hepatic inflammation, and insulin resistance of NAFLD rodents^28–30^. However, it remains unclear whether gut microbiota affects the therapeutic effect of OCA treatment on NAFLD. Here, the present study was conducted to decipher the dynamic complexity of the gut microbiota-bile acids axis during OCA treatment in NAFLD on mice by integrating metagenomics and metabolomics approaches. This study could lay a novel theoretical foundation for the treatment of NAFLD by OCA in the future.

## Results

### Gut microbiota plays a critical role in the treatment of OCA in NAFLD mice

**Fig. 1:**
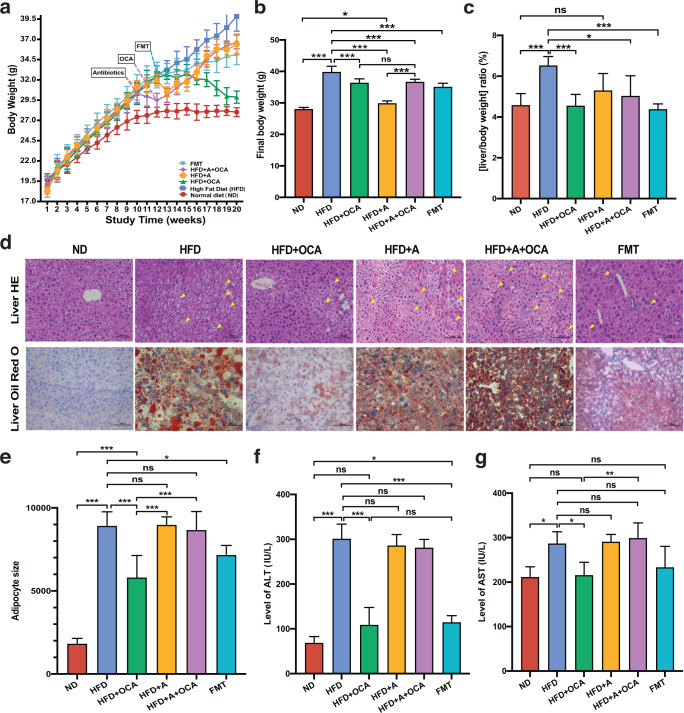
HFD induced NAFLD in mice and the therapeutic effect of different treatments. **a** Body weight curves of mice. **b** Body weight of mice at the last week (week 20) of the experiment. **c** Ratio of liver weight to body weight in mice. **d** Representative histological images of HE-stained and Oil red O-stained liver. **e** The adipocyte size of HE-stained adipose tissue in different groups of mice. **f** The level of ALT in the serum of mice. **g** The level of AST in the serum of mice. ND: group of normal diet-fed mice; HFD: group of HFD-fed mice; AIMD/ + A: group of HFD-fed mice with antibiotics intervention; +OCA: group of HFD-fed mice treated following OCA treatment; +A + OCA: group of HFD-fed mice treated with antibiotics intervention and following OCA treatment; FMT: group of HFD-fed mice transplanted with fecal microbiota of HFD + OCA. T-test, *P < 0.05; **P < 0.01; ***P < 0.001. ns: no significant difference. The data are presented as means ± SD.

High-fat-diet feeding had a great influence on the body weight of mice. Compared with the mice fed with normal diet (ND group), the body weight of mice fed with high-fat-diet (HFD group) was consistently significantly increased (*P* < 0.01, T-test) from the fourth week to the end of the experiment (Fig. 1a, b). OCA treatment to the HFD mice (HFD + OCA group) could apparently decrease (*P* < 0.01, T-test) the body weight, besides, both mice with 10 weeks of HFD feeding followed by 2 weeks of antibiotic-induced microbiome depletion (AIMD) (HFD + A group) and mice subsequently treated with OCA (HFD + A + OCA group) had notably decreased body compared with HFD mice (Fig. 1a, b). In addition, HFD mice transplanted with fecal microbiota from HFD mice with OCA treatment (FMT group) also exhibited a significant decline in the body weight compared to HFD mice. It was noteworthy that the body weight of all the groups of mice fed with HFD was significantly increased compared with ND group, even the HFD + OCA group which had the minimum body weight (Fig. 1b). No difference was observed in the food intake of mice among the groups of HFD, HFD + OCA, HFD + A + OCA, and FMT during this experiment, and food intake in the groups of mice fed with HFD was significantly declined compared with ND mice (Supplementary Figure 1a). The ratio of liver to body weight in HFD mice was significantly higher than that in ND mice, AIMD, OCA treatment, and FMT could decrease the elevated ratio in HFD mice and recover it to a comparable level in ND mice, although only a trend to decrease was found in HFD + A mice compared with ND mice (Fig. 1c). Meanwhile, hematoxylin-eosin (HE) staining of liver tissues of mice showed that HFD could exert histopathological effects on the liver, HFD, HFD + OCA, HFD + A, HFD + A + OCA, and FMT groups had hepatocellular ballooning, and dramatically increased size of hepatocytes and nuclear marginalization compared to ND mice, OCA treatment and FMT could effectively alleviate the liver lesion to some extent (Fig. 1d). In oil red O staining of mouse liver tissues (Fig. 1e), lipid droplets were obviously visible in hepatocytes of mice in HFD, HFD + A, and HFD + A + OCA groups, indicating that liver fat accumulation was serious in these three groups. In addition, HE staining of mouse adipose tissues showed that the adipocyte size in all the groups of mice fed with HFD (e.x. HFD, HFD + A, HFD + A + OCA, FMT) were significantly larger than ND mice, and adipocyte size in HFD group was similar with HFD + A and HFD + A + OCA groups, however significantly larger than OCA treatment and FMT groups (Fig. 1e, Supplementary Figure 1b). Hence, both OCA treatment and FMT could effectively alleviate liver and adipose histopathological changes caused by HFD in mice, although less significant effects exerted by FMT. In other words, gut microbiota played a critical role in the treatment of OCA on NAFLD induced by HFD in mice.

Apart from the changes in histopathological phenotypes of liver and adipose tissues, the liver function of mice was impaired by HFD compared to ND mice, and OCA treatment and FMT could greatly recover the injured liver function in HFD mice. Compared with ND mice, the levels of serum ALT and AST in HFD mice were dramatically increased, while OCA treatment and FMT could effectively reduce the elevated levels in HFD mice (Fig. 1f, g). Besides, HFD induced the glucose intolerance and insulin resistance with significantly increased levels of fasting serum insulin, blood glucose, and homeostatic model assessment for insulin resistance (HOMA-IR), and these adverse effects can be mitigated by OCA treatment and FMT (Supplementary Figure 1c, e). Similarly, OCA apparently reduced the levels of total triglyceride (TG), total cholesterol (TC), and low-density lipoprotein cholesterol (LDL-C), while increasing the level of high-density lipoprotein cholesterol (HDL-C) in the serum of HFD mice, less significant effects were produced by FMT (Supplementary Figure 1f–i). As for inflammatory factors, HFD induced increased levels of serum IL-6 and IL-1β in the mice, and no difference between HFD and ND mice was noticed in the level of TNF-α, only OCA treatment effectively reduced the levels of serum IL-6 and IL-1β in HFD mice (Supplementary Figure 2a–c). The intestinal barrier function was evaluated by the expression of occludin in the ileum tissue, HFD significantly decreased the expression of occludin expression in HFD, and only OCA treatment was adequate to recover to the reduced level of occludin in HFD (Supplementary Figure 2d, e). These results suggested that OCA treatment and FMT could alleviate liver and adipose tissue lesions caused by HFD, and improve liver function and intestinal barrier function, however the effectiveness of OCA treatment on NAFLD depends on the gut microbiota.

### The influence of OCA treatment on gut microbiota composition of NAFLD mice

**Fig. 2:**
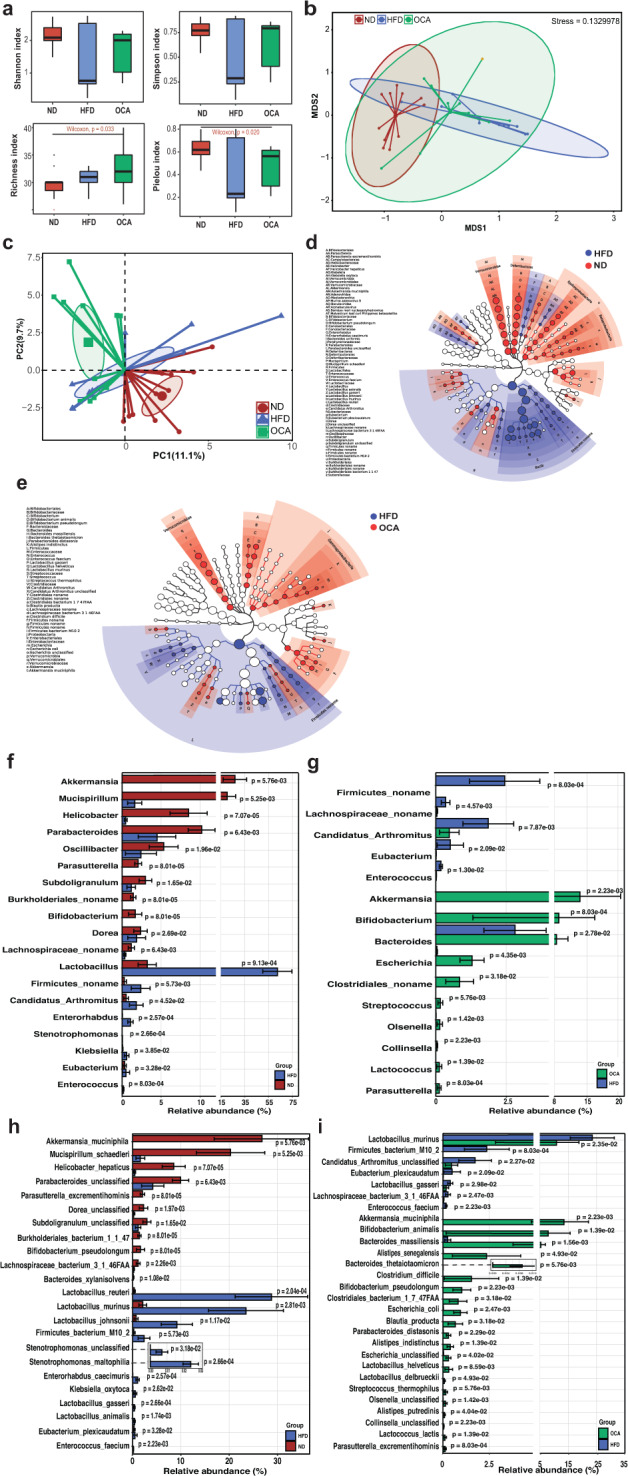
Gut microbiota profiling in the mice of ND, HFD, and OCA treatment to HFD mice. **a** Alpha diversity of microbial communities in ND, HFD, and OCA groups, including Shannon index, Simpson index, Richness index, and Pielou evenness. **b** Coordination plot of non-metric multidimensional scaling (NMDS) results of microbial communities among ND, HFD, and OCA groups based on Bray–Curtis dissimilarity. **c** Coordination plot of principal component analysis (PCA) of microbial communities among ND, HFD, and OCA groups. **d**, **e** The cladograms of differentially abundant taxa (according to LEfSe) between groups, **d** ND vs. HFD and (**e**) HFD vs. OCA. **f**, **g** Differential gut microbiota composition at genus level between groups, **f** ND vs. HFD, **g** HFD vs. OCA. **h**, **i** The differences of gut microbial composition at species level between groups, **h** ND vs. HDF, **i** HFD vs. OCA. ND, the group of normal-diet-fed mice; HFD, the group of high-fat-diet-fed mice; OCA, the group of mice treated with high-fat diet feeding and following OCA treatment. The data are presented as means ± SD.

To investigate the specific gut microbes involved in OCA treatment on NAFLD, we further implemented metagenomic analysis on the gut microbiota of ND, HFD, and OCA mice. For alpha diversity, Shannon index and Simpson index of gut microbiota had only consistent trends and no significant difference among the three groups of mice (Fig. 2a). However, OCA treatment substantially improved the richness of gut microbiota in HFD-fed mice. In addition, different Pielou indexes indicated that the highest gut microbiota evenness in mice of normal diet (ND) group, while lowest in mice of HFD group. The slightly elevated Pielou index suggested a higher evenness of the gut microbiota in OCA group compared with HFD group. For the gut microbial community structures, both non-metric multidimensional scaling (NMDS) based on Bray–Curtis dissimilarity distance matrix and principal component analysis (PCA) at species level were implemented, the results exhibited significant differences in microbial communities among ND, HFD, and HFD + OCA groups (adonis *R*^*2*^ = 0.243, *P* = 0.001, permutational multivariate ANOVA) (Fig. 2b, c). Therefore, gut microbiota structures of mice could be affected by both 20-week HFD feeding and HFD feeding with subsequent 8-week OCA treatment.

Further exploration of the gut microbial composition at different taxonomic levels, significant differences were found between HFD group and ND group, as well as OCA group and HFD group. At the phylum level, fecal microbiota mainly consisted of four phyla of bacteria: Firmicutes, Bacteroidetes, Proteobacteria, and Verrucomicrobia (Supplementary Figure 2f). The comparisons of the proportions of these four kinds of bacteria among the three groups demonstrated that HFD substantially increased the proportion of Firmicutes compared to ND mice (*P* < 0.001, Wilcoxon rank-sum test), while decreased the proportions of Proteobacteria and Verrucomicrobia (*P* < 0.01, Wilcoxon rank-sum test). In contrast, OCA not only profoundly reduced the proportion of *Firmicutes* (*P* < 0.01, Wilcoxon rank-sum test), but also improved the proportions of Proteobacteria (*P* < 0.01, Wilcoxon rank-sum test) and Verrucomicrobia (*P* < 0.01, Wilcoxon rank-sum test) in HFD group.

At the genus level, compared with HFD group, the enriched bacteria of mouse gut in ND group were *Akkermansia*, *Mucispirillum*, *Helicobacteria*, *Parabacteroides*, *Oscillibacter*, *Parasutterella*, *Subdoligranulum*, *Bifidobacterium*, and *Dorea* (Fig. 2d, f). And bacteria enriched in OCA group compared with HFD group were nine genera including *Akkermansia*, *Bifidobacterium*, *Bacteroides*, *Escherichia*, *Streptococcus*, *Olsenella*, *Collinsella*, *Lactococcus*, and *Parasutterella* (Fig. 2e, g). At the species level, *Akkermansia muciniphila*, *Bifidobacterium animalis*, *Bacteroides massiliensis*, *Alistipes senegalensis*, *Bacteroides thetaiotaomicron*, *Bifidobacterium pseudolongum*, *Escherichia coli*, *Blautia producta*, *Parabacteroides distasonis*, *Alistipes indistinctus*, *Lactobacillus helveticus*, *Lactobacillus delbrueckii*, *Streptococcus thermophilus*, *Lactococcus lactis*, and *Parasutterella excrementihominis* exhibited higher abundance in OCA group compared to HFD group (Fig. 2i). Also, OCA intervention significantly reduced the proportions of *Lactobacillus murine*, *Firmicutes bacterium* M10-2, *Lactobacillus gasseri*, and *Enterococcus faecium* in the gut of HFD-fed mice. Besides, compared with HFD group, ND group had a higher abundance of *A. muciniphila*, *Mucispirillum schaedleri*, *Parasutterella excrementihominis*, *B. pseudolongum*, *Bacteroides uniformis*, *Bacteroides xylanisolvens*, and lower abundance of eleven species including the same four species reduced by OCA treatment (Fig. 2h). Thus, OCA treatment recovered the gut microbial composition that was altered by HFD, and made it approximate to that in ND group.

### The bile acid profiles and correlations between bile acid and gut microbiota

**Fig. 3:**
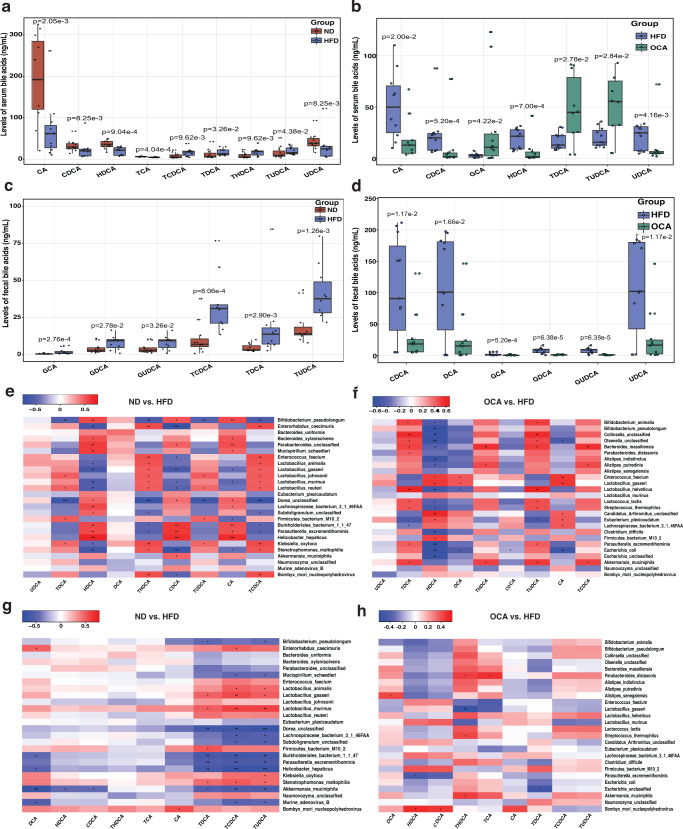
Bile acids composition in ND, HFD, and OCA groups and correlations between the abundance of bile acids and gut microbes in mice. **a**, **b** Levels of serum bile acids in the mice between groups, **a** ND vs. HFD, **b** HFD vs. OCA. **c**, **d** Levels of fecal bile acid in the mice between groups, **c** ND vs. HFD, **d** HFD vs. OCA. **e**, **f** Correlations between gut microbes and serum bile acids in the mice between groups, **e** ND vs. HFD, **f** HFD vs. OCA. **g**, **h** Correlations between gut microbes and fecal bile acids in mice between groups, **g** ND vs. HFD, **h** HFD vs. OCA. HFD, the group of high-fat-diet-fed mice; ND, the group of normal-diet-fed mice; OCA, the group of mice treated with high-fat diet feeding and following OCA intervention. Red grids represented the positive correlation; blue grids represented the negative correlation; BA: bile acid. Wilcoxon rank-sum test and Spearman’s rank correlation, *P < 0.05; **P < 0.01; ***P < 0.001. In the box plots, horizontal line within the rectangle represents the median of all values, The top end of the box represents the upper quartile (75%), while the bottom end of the box represents the lower quartile (25%). The data are presented as means ± SD in the bar plots.

It is well-known that gut microbiota is tightly associated with bile acid metabolism, shifts in gut microbiota might influence the host bile acids pool. Indeed, targeted metabolomics analysis of bile acids indicated that both HFD and HFD with following OCA treatment could effectively modify the serum bile acid pool in mice. Compared with ND group, HFD feeding profoundly increased the level of conjugated bile acids, taurochenodeoxycholic acid (TCDCA), taurohyodeoxycholic acid (THDCA), and TUDCA (HFD vs. ND, 2.8 times higher), and decreased the levels of unconjugated bile acids, such as CA and CDCA in the mice serum (Fig. 3a). In contrast, OCA significantly reduced the levels of CA, CDCA, hyodeoxycholic acid (HDCA), and ursodeoxycholic acid (UDCA) in HFD mice (Fig. 3b). Different variations were observed in the levels of fecal bile acids among groups, HFD apparently increased (*P* < 0.05, Wilcoxon rank-sum test) the levels of most fecal bile acids in mice compared with ND group (Fig. 3c), while OCA decreased the amounts of most fecal bile acids increased by HFD feeding including the level of fecal conjugated bile acids of glycodeoxycholic acid (GDCA) and glycoursodeoxycholic acid (GUDCA) (Fig. 3d).

To analyze whether bile acids variations in serum and feces were driven by gut microbes, we performed Spearman correlation analysis on the abundance of species-level of bacteria and level of bile acids among the ND, HFD, and OCA groups. Generally, variations of serum bile acids were stronger correlated with gut microbiota than serum bile acids variations. Bacteria species of *B. massiliensis*, *A. muciniphila*, *L. johnsonii*, and *L. reuteri* were positively correlated with the levels of serum TCDCA, THDCA, and TUDCA, and were significantly negatively correlated with the level of HDCA except for *A. muciniphila* (Fig. 3e, f). Besides, *E. coli* exhibited a significantly negative correlation with the levels of serum HDCA, CDCA, and CA (Fig. 3f). Meanwhile, significant correlations between mouse gut microbiota and fecal bile acids were also obtained. The abundance of two species of *Lactobacilli* (*L. murinus* and *L. gasseri*) exhibited significant positive correlations with the level of fecal TDCA, TUDCA, and TCDCA, while *A. muciniphila* was negatively correlated with fecal TCDCA, THDCA, and TUDCA (Fig. 3g). In addition, *B. pseudolongum* showed negative correlations with both the serum and fecal TDCA and TUDCA (Fig. 3g). Both *A. muciniphila* and *S. thermophilus* displayed positive correlations with fecal THDCA, while *L. gasseri* was vice versa (Fig. 3h).

### The potential of bacterial genomes encoding enzymes involved in bile acids metabolism

**Fig. 4:**
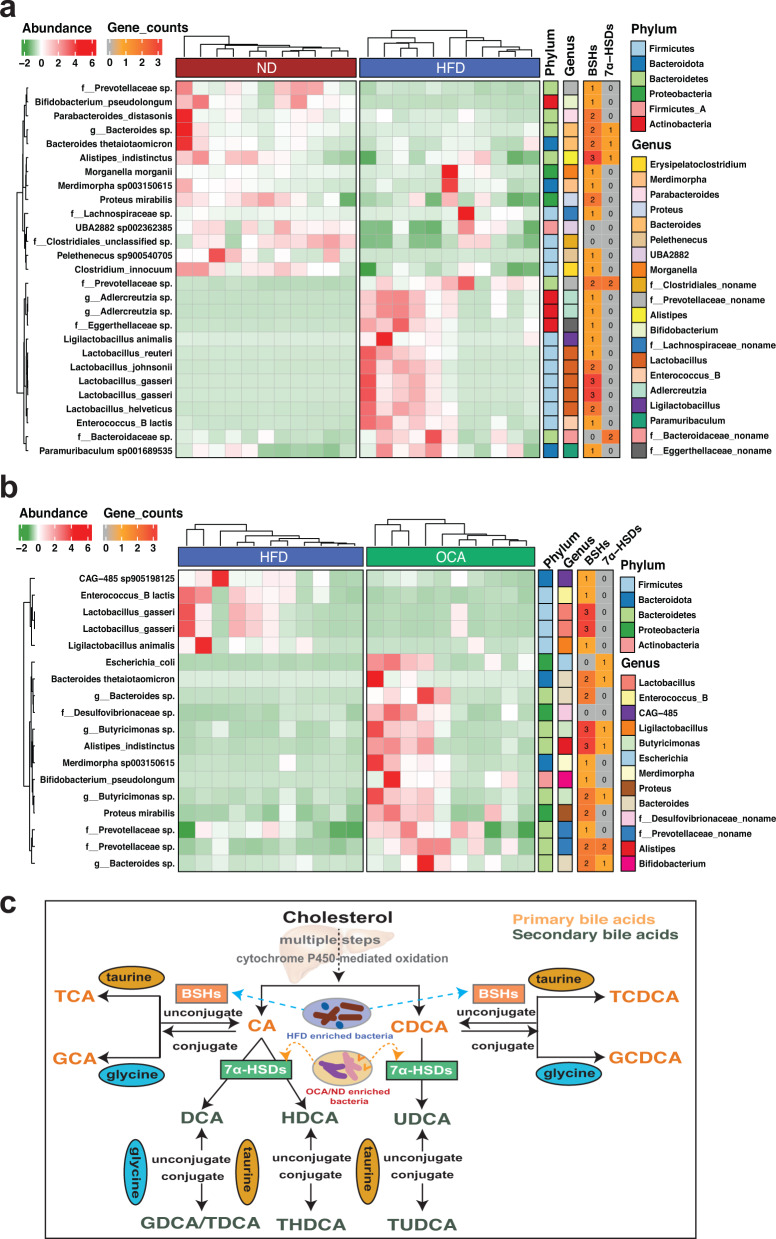
Enzymes screening of specially enriched gut microbes in ND, HFD, and OCA groups based on metagenome-assembled genomes. **a** Differentially abundant SGBs in ND vs. HFD groups, and the potential of group-enriched SGBs in encoding enzymes of BSHs (EC:3.5.1.24) and 7α-HSDs (EC:1.1.1.159). **b** Differentially abundant SGBs in OCA vs. HFD groups, and the potential of group-enriched SGB in encoding enzymes of BSHs and 7α-HSDs. **c** Diagram of the bile acids transformations and the potential role of group-enriched gut microbes in the modification of bile acids.

To further investigate the functional profiles of gut microbiota influencing bile acids metabolism, we performed enzymes scanning based on the species-level genome bins (SGBs, see Methods) enriched in different groups (HFD vs. ND, HFD vs. OCA). Compared with HFD group, SGBs mainly enriched in ND were species of *Bacteroides*, *Parabacteroides*, *and Prevotellaceae* in *Bacteroidetes*, *Bifidobacterium pseudolongum*, *Alistipes indistinctus*, and *Clostridium innocuum* (Fig. 4a). And OCA treatment significantly elevated the abundance of two species of *Bacteroides*, *Bifidobacterium pseudolongum*, *Alistipes indistinctus*, two species of *Prevotellaceae*, and *Escherichia_coli* and *Proteus mirabilis* belonging to *Proteobacteria* (Fig. 4b). Worthy of note was that HFD compared to OCA treatment enriched bacteria exclusively belonging to *Firmicutes* (Fig. 4b). Intriguingly, some bacteria species were both enriched in ND and OCA, such as *Bifidobacterium pseudolongum*, *Alistipes indistinctus*, and species of *Bacteroides* and *Prevotellaceae*. The enzymes encoded by SGBs enriched in different groups indicated that bacterial species enriched in ND and OCA groups had the adequate potential to encode both the BSHs (EC:3.5.1.24) and 7α-HSDs (EC:1.1.1.159), while HFD enriched bacteria exhibited poor capacity of encoding enzymes of 7α-HSDs and mainly encoded BSHs responsible for primary bile acids transformation (Fig. 4a, b, Supplementary Table 3). Collectively, HFD enabled the accumulation of bacteria encoding BSHs which accelerated the secretion of bile into the gut and the uncoupling of conjugated bile acids into primary bile acids. And OCA treatment elevated the abundance of bacteria with the potential to encode 7α-HSDs which facilitated the degradation of primary bile acids and the promotion of secondary bile acids bioconversion in the gut (Fig. 4c).

## Discussion

NAFLD, a multifactorial-related disease with complex pathogenesis, is becoming the predominant manifestation of chronic liver diseases with a global prevalence of about 25%^31–33^. Although studies on human^34,35^ and mouse models^36,37^ provided evidence of a causal role of gut microbiota in NAFLD development, knowledge gap still exists in whether gut microbiota influences the therapeutic action of NAFLD medication. The present study explored the role of gut microbiota on the therapeutic effect of OCA treatment on NAFLD induced by the high-fat diet. In summary, we first confirmed the critical role of gut microbiota on NAFLD therapeutic effects and identified the key microbes modulating the host bile acids pool thereby contributing to NAFLD development and OCA therapeutic effects of NAFLD. Our study could provide new insights into the bile acids metabolism regulation by the gut microbes during NAFLD development and treatment and lay the theoretical foundation for NAFLD prevention and treatment through gut microbiota interventions.

In the previous studies, OCA was found to impressively mitigate hepatic lipid accumulation, liver inflammation, and insulin resistance in NAFLD mice^28,38,39^. However, the effect of gut microbiota on OCA treatment for NAFLD remains unclear. The current study implemented AMID and FMT trials and confirmed the critical role of gut microbial community during NAFLD treatment by OCA. Similar results were reported in studies that treated NAFLD with antibiotics^40^ and probiotics^41,42^ in which bacteria species were supplemented as probiotics and improved liver function of NAFLD. Compared with HFD group, both ND and OCA enriched the common genera including *Akkermansia*, *Bifidobacteria*, and *Bacteroides* which exactly comprised varieties of beneficial microbes. Indeed, Within the three genera, two probiotic species of *A. muciniphila* and *B. pseudolongum* were both attractively enriched in OCA and ND. *A. muciniphila*, a gut microbial member in healthy individuals, was found to exhibit host immunoregulatory effects^43^ and could reverse the metabolic disorders in high-fat diet-induced obesity and type 2 diabetes^44^, while *B. pseudolongum* was considered effective to improve the lipid metabolism of obese mouse model^45^. AIMD and FMT confirmed that gut microbiota was essential in the therapy of NAFLD, and the differential gut microbial compositions suggested that specific microbes were involved in NAFLD development and therapy.

Bile acids and their receptors have emerged as important regulators of hepatic lipid metabolism^46^, and microbial modification of bile acids is an important mechanism by which the microbiota can interact with the host and affect liver disease^9^. Our results showed that HFD increased the levels of serum-conjugated bile acids (e.g., TCDCA, THDCA), which might increase the paracellular permeability and lead to gastrointestinal diseases^47^. And OCA obviously decreased the levels of primary bile acids (e.g., CA and CDCA) in mouse serum, most free bile acids (e.g., DCA, CDCA, UDCA) and conjugated bile acids (e.g., TDCA, TUDCA) in mouse feces. The primary bile acids, like CA and CDCA, were able to disrupt membranes and cause intracellular damage^48^. Our finding is consistent with the previous study^49^ suggesting that OCA was able to inhibit atherosclerosis by ileum bile acids metabolism modulation. In terms of bile acids, serum metabolites in circulation are usually better in the manifestation of host metabolism and determine the progression of diseases. Noticeably, in the present study, serum TUDCA was predominantly increased in OCA group (2.8 times higher than HFD group). Meanwhile, OCA group enriched species such as *Akkermansia muciniphila*, *Bifidobacterium animalis*, *Bacteroides massiliensis*, and *Lactobacillus helveticus* which were significantly positively correlated with serum TUDCA. Less and weaker correlations were found between gut microbes and fecal bile acids in the present study. These results suggested that serum bile acids were more sensitive to the alteration of gut microbial alterations. Chen et al.^50^ reported circulating bile acids rather than fecal bile acids showing apparent variations along with liver disease development which suggested circulating bile acids as promising indicators of disease status. Our study further indicated the difference in circulating and fecal bile acids was probably associated with gut microbiota alterations. More evidence was provided by functional profiling of metagenome-assembled genomes, those species enriched in ND and OCA groups had the potential in encoding enzymes of BSHs (*Bacteroides* spp., *Alistipes* spp., and *Bifidobacterium* spp.) and 7α-HSDs (e.g., *Alistipes* spp. and *Bacteroidaceae* species) which were responsible for bioconversion of primary and secondary bile acids. These results demonstrated that the therapeutic effects of NAFLD by OCA could be attributed to the modification of host bile acids by special gut microbes. Communication between the liver and intestine can be mediated by bile acids which are ligands for the nuclear receptor farnesoid X receptor (FXR) and the G-protein–coupled receptor TGR5^49^. FXR, as a ligand-activated nuclear receptor, plays an important role in repressing lipogenesis, promoting FA oxidation, and reducing fatty acids uptake in liver^51^. And TGR5 could exert a capacity to maintain glucose homeostasis and inhibit inflammation, thus improving NAFLD features^52^. The present study confirmed the pivotal role of gut microbiota in NAFLD treatment by OCA and revealed differential bile acids profiles resulting from gut microbial alterations caused by the high-fat diet and the additional OCA treatment.

In conclusion, gut microbes, especially *Bacteroides* spp., *Alistipes* spp., and *Bifidobacterium* spp., could interact with the host through bile acids modification by encoding enzymes of BSHs and 7α-HSDs, thus determining the therapeutic effect of NAFLD treatment by OCA. Although the causality of gut microbiota to NAFLD therapeutic effects was confirmed in the current study, the mechanism by which variation in specific bile acids contributes to NAFLD therapeutic effects by OCA still requires further exploration. Future studies are still required to specifically decipher the interactions between gut microbiota and host bile acid metabolism, as well as how bile acids affect the development and treatment of NAFLD.

## Methods

### Animal and treatments

**Fig. 5:**
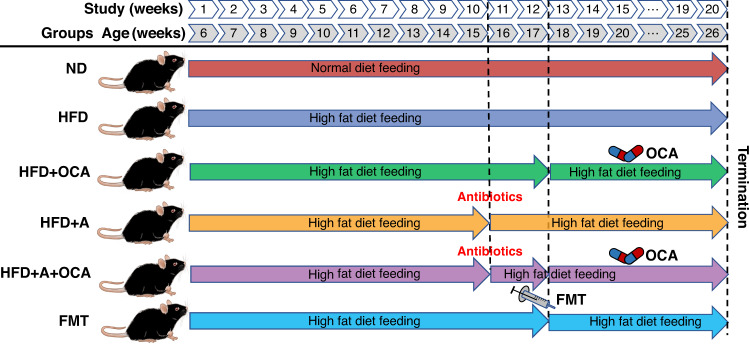
Experiment design of the NAFLD study on mice. Each group consists of 11 randomly assigned male mice of 6-week-old. ND: group of mice fed normal diet for 20 weeks; HFD: group of mice fed HFD for 20 weeks; HFD + OCA: group of mice fed HFD for 12 weeks and following 8 weeks of OCA treatment; HFD + A: group of mice fed with HFD for 10 weeks and following 2 weeks of antibiotics intervention; HFD + A + OCA: group of 10 weeks of HFD feeding mice treated with 2 weeks of antibiotics intervention and following 8 weeks of OCA treatment; FMT: group of 12 weeks of HFD feeding mice transplanted with fecal microbiota of HFD + OCA.

Six-week-old specific-pathogen-free male C57BL/6J mice were provided by the Department of Laboratory Animals, Kunming Medical University. Sixty-six mice were adopted, divided equally into six groups (each group, *n* = 11), and randomly assigned to the normal diet (ND, as control), high-fat diet (HFD), HFD + OCA (HFD following with OCA treatment), HFD + A (HFD following with antibiotic intervention), HFD + A + OCA (HFD followed by antibiotic-induced microbiome depletion and then OCA treatment), and FMT groups, the whole experimental period for each group was 20 weeks (Fig. 5). OCA treatment in this study was implemented with oral gavage of OCA (50 mg/kg body weight) once a day. Fecal microbiota transplantation (FMT) was performed from the mice of HFD + OCA (as donors) to the mice fed with 12 weeks of HFD (as recipients). Each group in this study consisted of eleven mice, all mice were housed in a temperature- and humidity-controlled environment at Kunming Medical University with a 12 h/12 h light/dark cycle, with free access to food and water. All animal studies and experimental procedures were approved by the Ethics Committee of Kunming Medical University (license No. kmmu2021154).

### NAFLD model establishment and antibiotic-induced microbiome depletion

The high-fat diet (HFD) induced NAFLD mouse models were established with 12 weeks of HFD feeding to mice after a normal adaptive feeding except for HFD + A + OCA group (10 weeks of HFD feeding). We referred to the reported protocol for preparing an antibiotic cocktail (ampicillin 1 g/L + vancomycin 500 mg/L + neomycin 1 g/L + metronidazole 1 g/L)^53^. Mice in HFD + A + OCA were fed with HFD for 10 weeks, the mice received antibiotic cocktail in drinking water for 2 weeks. At week 12, the mice were given oral gavage of OCA (50 mg/kg body weight) for 8 weeks once a day. The indexes for detection were body weight alternation, glucose tolerance, insulin resistance, total cholesterol, triglyceride, hepatic fat accumulation, impaired intestinal barrier function, and so on.

### Fecal microbiota transplantation (FMT)

For the FMT group, After HFD was given to mice for 12 weeks, FMT was initiated and performed for 8 weeks (the total period was 20 weeks). Fresh fecal samples were collected from mice of HFD + OCA. These collected fecal samples were diluted with saline solution and vigorously vortexed for 1 min. Then the homogeneous liquids were centrifuged, and the supernatants were kept for FMT treatment. Fresh supernatants regarded as graft materials were prepared within 10 min before oral gavage for preventing variations in microbiota composition. During the experiment, the body weight of mice in each group was monitored weekly, and the manifestations as well as the food intake dose of mice were documented once a week. Meanwhile, the related biochemical and pathological examinations of mouse serum and liver tissue were also performed weekly.

### Histological analysis

Mouse liver tissues were rinsed in general stationary liquid and fixed for over 24 h. After fixation, tissues were dehydrated in 30% sucrose for over 48 h The dehydrated tissues were embedded at −25 °C, and then the frozen tissues were sectioned into slices with 8–10 μm thickness. Next, these slides were incubated in Oil Red O working solution for 8–10 min away from light, and the specific staining time depended on the actual situation. After the slight air drying, the dyed slides underwent differentiation in 60% isopropanol solution, followed by hematoxylin counter-staining for 3–5 min. The staining time also relied on the actual situation. After washing under running water and differentiation in isopropanol, the slides were rinsed in bluing buffer, and then they were rinsed in running water again. Finally, these slides were mounted with glycerine jelly and covered by coverslips. The liver tissue and adipose tissue were first fixed and embedded in paraffin, sectioned (5 μm) and stained with hematoxylin and eosin by standard procedures. The quantification of adipocyte size was performed using ImageJ^54^.

### Examination of biochemical indexes, inflammatory factors, and intestinal barrier function

We collected a 500 μl mouse blood sample, which was followed by 2000 × *g* centrifugation for 10 min. Next, the supernatant was kept for detecting biochemical indexes by the automatic biochemical analyzer (Cobas 6000 c501; Roche Diagnostics, Basel, Switzerland), such as the levels of TC, TG, ALT, AST, triglyceride, high-density lipoprotein (HDL), low-density lipoprotein (LDL), fasting blood glucose and insulin. The levels of serum inflammatory factors (IL-6, IL-1β, and TNF-α) were determined using a commercial ELISA kit (IL-6, YFXEM00045; IL-1β, YFXEM00028; TNF-α, YFXEM00031, Yfxbio, Nanjing, China) following the manufacturer’s instructions. The intestinal barrier function was evaluated by the protein expression of occludin in the ileum tissue using Western Blot. Specifically, ileum tissue was homogenized in RIPA buffer. Proteins were separated using 10 or 12.5% sodium dodecyl sulfate polyacrylamide gel electrophoresis (SDS-PAGE) (PG112, PG112; Yase company, Shanghai, China), and transferred to polyvinylidene fluoride (PVDF) membranes before being blocked for 2.5 h with 5% skim milk-TBST. The following antibodies were incubated with the blots: anti-Occludin (#DF7504; 1:1000); Affinity Biosciences, China), and anti-GAPDH (1:2000; #97166; Cell Signaling Technology, Beverly, MA, USA). After subsequent incubation with secondary antibodies, the blots were imaged using an imaging system (Amersham Imager 600) and ECL substrate reagents (#32109, Thermo Scientific Science, Waltham, MA, USA). The gray values in ImageJ were used for quantification. All gels were processed in parallel and derived from the same experiment.

### Fecal metagenomic sequencing and bioinformatic analysis

#### Shotgun metagenomic sequencing for fecal samples

At the end of the experiment, we separately collected fresh fecal contents from mice in the ND group, HFD group, and OCA-intervened HFD (OCA) group, group information for individual assignment can be found in Supplementary Table 1. These samples were stored at −80 °C until DNA extraction. DNA quality and quantification were evaluated with a NanoDrop Spectrophotometer ND-1000 (Thermo Fisher Scientific). Metagenomic DNA libraries were constructed according to the instructions of NEXTflex Rapid DNASeq Kit and were 150-bp paired-end sequenced on the Illumina NovaSeq 6000 platform (Shanghai Biotechnology Corporation).

#### Bioinformatic analysis for metagenomic data

Quality control of raw data was performed with FastQC (v 0.11.8) (https://www.bioinformatics.babraham.ac.uk/projects/fastqc/) and Trimmomatic (v 0.32)^55^ with parameters of “SLIDINGWINDOW:4:20 MINLEN:50”, and bowtie2 (v 2.3.1)^56^ was aligned to genome reference consortium mouse Build 38 (GRCm38) to remove host genomic sequences. High-quality microbial reads were de novo assembled into contigs using metaSPAdes (SPAdes-3.10.1)^57^. Then, open reading frames (ORFs) were predicted from the assembled contigs with MetaGeneMark (v 3.38)^58^ with default parameters. ORFs were clustered using CD-HIT (v 4.8.1)^59^ with 90% length with 95% identity to construct the non-redundant gene catalog. High-quality microbial reads were mapped to the non-redundant gene catalog to calculate the gene abundance using Salmon (v 1.3.1)^60^. Finally, the taxonomic assessment was performed using MetaPhlAn (v 2.1.0)^61^ and functional annotation was conducted using DIAMOND (v 0.9.7.108)^62^ against eggNOG (v 5.0)^63^ with “--more-sensitive” and “--evalue 1e-5”.

### Metagenomic analysis based on metagenome-assembled genomes and enzymes involved in bile acids metabolism of bacterial genomes

Metagenomic sequencing with binning strategy provides a better means to study the functional characteristics of specific gut microbes based on the genome level. Previous study^64^ on the human microbiome reconstructed over 150,000 metagenome-assembled genomes (MAGs) from different populations and recapitulated 4930 species-level genome bins (SGBs) by 95% similarity of average nucleotide identity of genomes, the considerable number of SGBs greatly expanded the current resource of reference genomes. To better explore the functional profile of the specific microbes involved in NAFLD, we took the 4930 SGBs as reference genomes and performed taxonomic and functional analyses based on the bacterial genomes. First, our high-quality microbial sequences were aligned to the reference genomes using BWA MEM (v0.7.17-r1188)^65^ and SAMtools (v 1.9)^66^ to calculate the abundance of SGBs in each sample, only SGBs with coverage of >40% were considered detected in the samples. The abundance of each SGB was computed as the depth of the contigs of SGBs normalized by the total length of the genome to allow for sample-to-sample comparison, the abundance of SGBs in ND, HFD, and OCA groups can be found in Supplementary Table 2. The differential abundant SGBs not being classified in the previous study were taxonomic annotated by GTDB-TK (v 2.1.0)^67^ against the Genome Taxonomy Database (GTDB release207_v2)^68^, and SGB-encoded proteins predicted by Prodigal (v.2.6.3)^69^ were annotated against KEGG database^70^ using DIAMOND (v 0.9.7.108, “--evalue 1e-5”) to screen the enzymes involved in bile acids metabolism.

### Targeted metabolomic analysis of bile acid in mice serum and fecal samples

The serum sample was mixed with the extract (methanol:acetonitrile = 5:3) in a ratio of 1:4. The supernatant was collected by centrifugation after full shaking and standing. The organic reagent was removed, and the supernatant was redissolved in 50% methanol for inspection; A 50 mg fecal sample was accurately weighed, and 6 µl of ice-cold methanol was added to each 1 mg sample. After grinding, the sample was crushed by shaking, standing, and centrifugation. A 1:6 solution of frozen methanol was added to the residue a second time and the procedure was repeated. The extract was combined twice, organic reagents were removed, and 200 µL 50% methanol was redissolved for later use. The instrument parameters used for sample loading (Instruments: Waters Acquity UPLC, mass spectrometry AB SCIEX 5500 QQQ-MS; Chromatographic column: Acquity UPLC BEH C18 (1.7 µm, 2.1 mm*100 mm)). The bile acid reference was weighed and a series of solutions containing the final concentration of the reference were prepared. The standard curve was drawn according to the peak area of different concentrations and the substandard of the corresponding concentrations. MultiQuant software (v 3.0.2, Sciex) was used for integration, and the content was calculated using the standard curve. The concentration of serum and fecal bile acids can be found in Supplementary Table 4.

### Statistical analysis

GraphPad Prism8.0 was used to cope with data acquired from the experiment, and these data were represented as mean ± SEM. According to circumstances, independent-sample T test or one-way ANOVA was performed for statistically comparative analysis of inter-group differences. Wilcoxon rank-sum test was conducted for inter-group comparison of the bile acid level. LEfSe^71^ was applied to the microbial composition analysis on different taxonomic levels, the cut-off of the LDA score was set to 2, and significant features were considered with *P*-values lower than 0.05. The exploration of the association between bile acid and gut microbiota was implemented by Spearman correlation analysis based on the Centered log-ratio (CLR) Normalized abundance. All the *P*-values were corrected using the Benjamini–Hochberg FDR method. Significant differences were recognized when adjusted-*P* values were less than 0.05. Most data are presented as mean ± SD.

## Supplementary information

Supplementary Information

Reportiing Summary

Supplementary Table 2

Supplementary Table 3

Supplementary Table 4

## Data Availability

Shotgun metagenomic sequencing data are available from Sequence Read Archive under bioproject ID PRJNA902833.

## Code availability

The workflow and detailed code to perform bioinformatic analyses are available on GitHub (https://github.com/ThomasYJK555/Zhanglab_NAFLD_study).
