# Supplementary material for: Gut microbiome determines therapeutic effects of OCA on NAFLD by modulating bile acid metabolism
Source: NPJ Biofilms Microbiomes. 2023 May 31;9:29. doi: 10.1038/s41522-023-00399-z (PMC10232493; doi:10.1038/s41522-023-00399-z)
Supplement: Supplementary file 1 — Supplementary Information [file 41522_2023_399_MOESM1_ESM.pdf]

Supplementary Materials for

Gut microbiome determines therapeutic effects of OCA on NAFLD by modulating bile acid metabolism

Jianjun Liu [1,2, 5], Jiayi Sun [3, 5], Jiangkun Yu[1,5,*], Hang Chen [4, 5], Dan Zhang [4], Tao Zhang[1], Yicheng Ma[1], Chenggang Zou[1], Zhigang Zhang[1,*], Lanqing Ma[4,*], Xue Yu[3,*]

[5]These authors contributed equally

*Correspondence: zhangzhigang@ynu.edu.cn (Z. Z.), 531229897@qq.com (L.M.), yuxuemd@aliyun.com (X. Y.), yjk555@ynu.edu.cn (J. Y.)

This PDF file includes:

Supplementary Figure 1 to 4

Other Supplementary Materials for this manuscript include the following:

Supplementary Table 1 to 4

Supplementary Figures

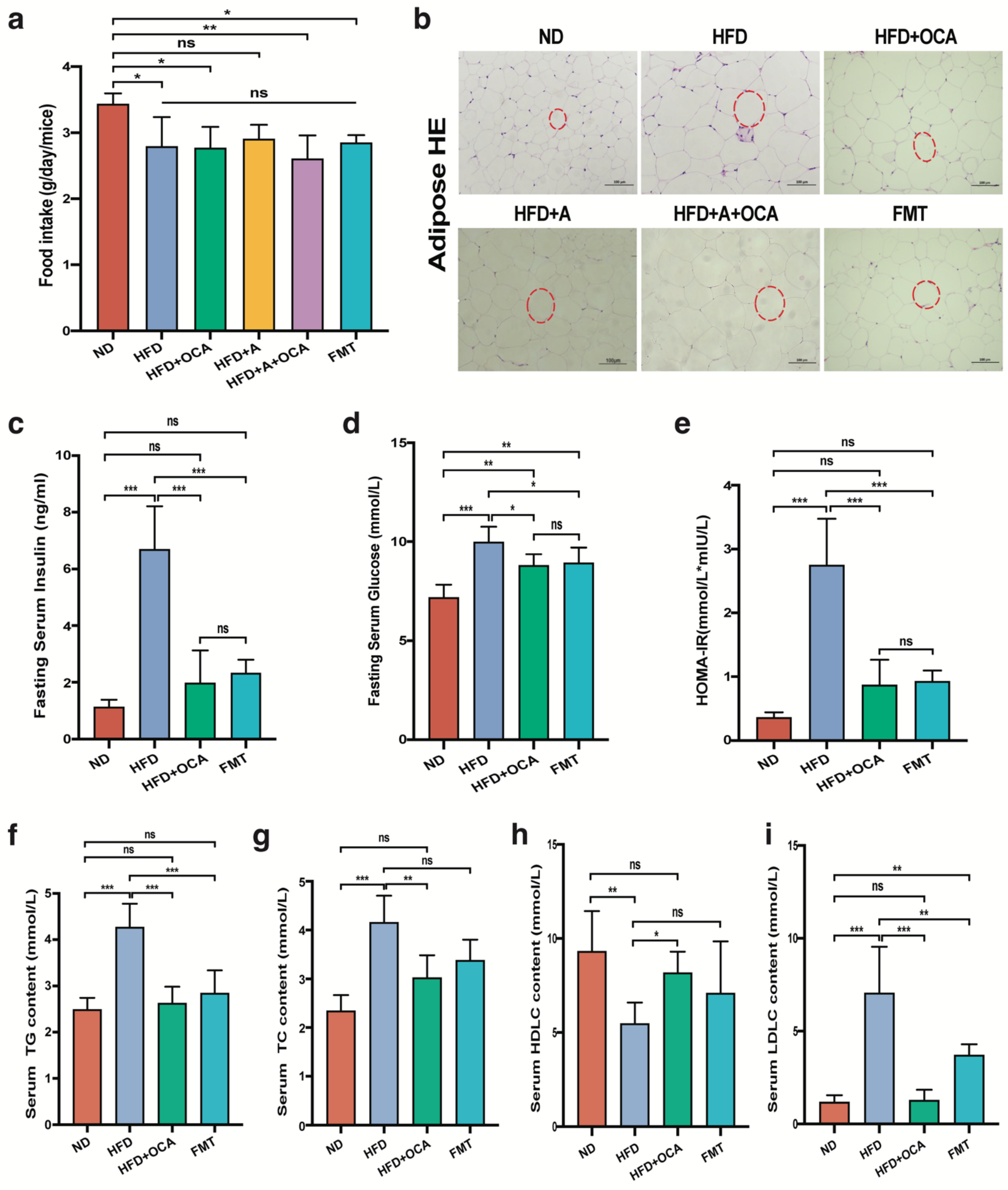

Supplementary Figure 1. Food intake, HE-stained adipose tissue, and the serum biochemical indexes examination of mice in different treatments.

(a) Average daily food intake of mice in all the groups of ND, HFD, HFD+OCA, HFD+A, and HFD+A+OCA. (b) Representative image of HE-stained adipose tissue showing the adipocyte size in different groups. (c-e) Comparisons of the biochemical indexes indicating glucose intolerance and insulin resistance, (c) The level of fasting serum insulin, (d) The level of fasting serum glucose, (e) The level of HOMA-IR. (f-i) Comparisons of the serum lipid related indexes. (f) Serum triglycerides (TG) content, (g) Serum total cholesterol (TC) content, (h) The level of serum high-density lipoprotein cholesterol (HDL-C), (i) The level of serum low-density lipoprotein cholesterol (LDL-C). ND: normal diet fed group; HFD: high-fat-diet group; +A: group of HFD-fed mice with antibiotics intervention; +OCA: group of HFD-fed mice treated following OCA treatment; +A+OCA: group of HFD-fed mice treated with antibiotics intervention and following OCA treatment; FMT: group of HFD-fed mice transplanted with fecal microbiota of HFD+OCA. T-test, *, $P < 0.05$; **, $P < 0.01$; ***, $P < 0.001$. The data are presented as means ± SD.

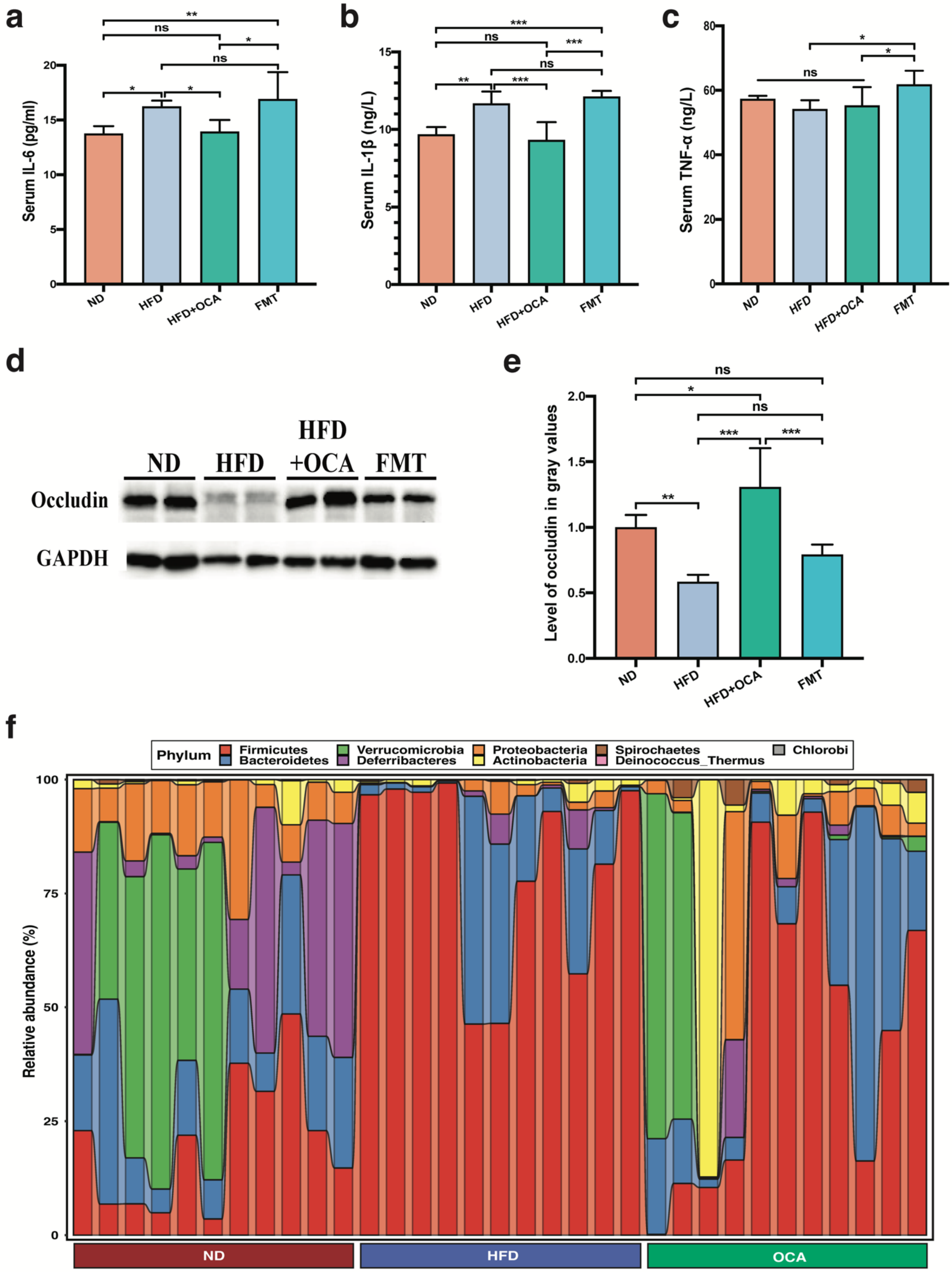
a
Serum IL-6 (pg/ml)
ND
HFD
HFD+OCA
FMT
b
Serum IL-1β (ng/L)
c
Serum TNF-α (ng/L)
d
ND
HFD
HFD +OCA
FMT
Occludin
GAPDH
e
Level of occludin in gray values
f
Phylum
Firmicutes
Bacteroidetes
Verrucomicrobia
Deferribacteres
Proteobacteria
Actinobacteria
Spirochaetes
Deinococcus_Thermus
Chlorobi
Relative abundance (%)
ND
HFD
OCA

Supplementary Figure 2. Profiles of serum inflammatory factors, intestinal function barrier, and microbial composition at the phylum level in different groups

(a-c) Profiles of serum inflammatory factors, (a) The level of serum IL-6 in mice of ND, HFD, HFD+OCA, and FMT groups, (b) The level of serum IL-1β in mice of ND, HFD, HFD+OCA, and FMT groups, (c) The level of serum TNF-α in mice of ND, HFD, HFD+OCA, and FMT groups. (d, e) Assessment of the expression of occludin in the ileum tissue of mice among ND, HFD, HFD+OCA, and FMT groups, (d) Western blot image shows the differential expression of *occludin* among groups (original blots can be found in Supplementary Figure 3 and 4), (e) The level of *occludin* in gray values of Western blot quantified by ImageJ. (f) Microbial composition at the phylum level in mice of ND, HFD, and OCA groups. ND, group of mice fed with normal diet; HFD, group of mice fed with high-fat-diet; OCA, group of HFD mice with following OCA intervention; FMT, group of HFD-fed mice received fecal microbiota transplantation from HFD-fed mice with OCA treatment. T-test, *, $P < 0.05$; **, $P < 0.01$; ***, $P < 0.001$. The data are presented as means ± SD.

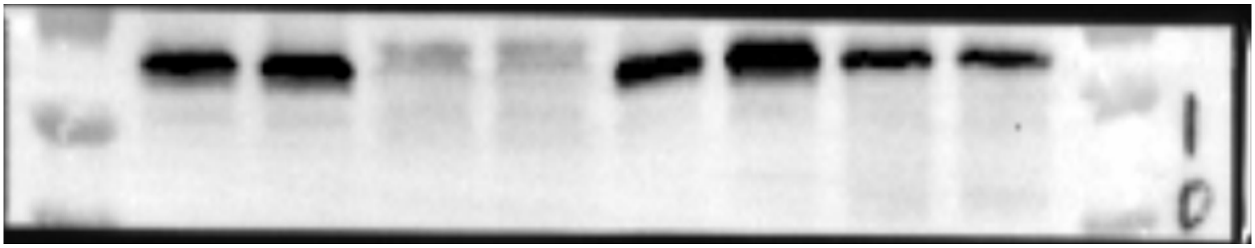

Supplementary Figure 3 Original blots of *occludin* (corresponding to Supplementary Figure 2d)

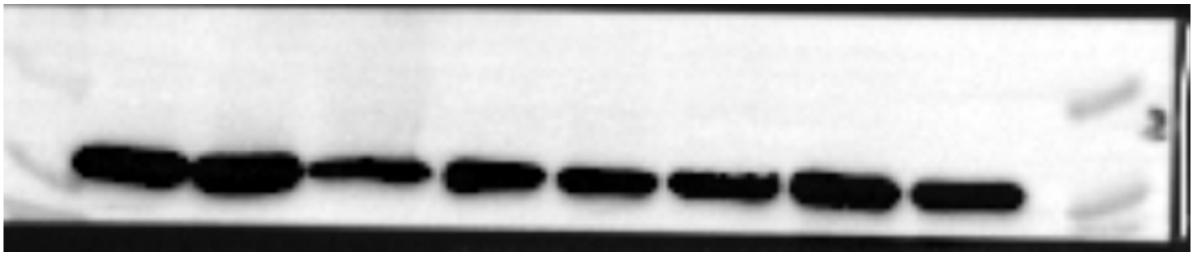

Supplementary Figure 4 Original blots of GAPDH (corresponding to Supplementary Figure 2d)

Supplementary Table 1 Group information of samples in the metagenomic study

Sample_ID	Sample size	Group_information
G11-G19, G110, G112	n = 11	Normal diet group (ND)
G31-G34, G37-G39, G310-G313	n = 11	High fat diet group (HFD)
G42-G49,<br>G410-G411	n = 10	High fat diet with following<br>OCA administration (OCA)

Supplementary Table 2 The abundance of reference SGBs

Supplementary Table 3 Enzymes encoding by differential SGBs in ND, HFD, OCA groups

Supplementary Table 4 Bile acids concentrations in the serum and fecal samples in ND, HFD, and OCA groups
